# Prognosis of COVID-19: Red Cell Distribution Width, Platelet Distribution Width, and C-Reactive Protein

**DOI:** 10.7759/cureus.13078

**Published:** 2021-02-02

**Authors:** Shivakumar Bommenahalli Gowda, Siddharth Gosavi, Amogh Ananda Rao, Shashank Shastry, Sharanya C Raj, Sanjana Menon, Ashutosh Suresh, Anirudha Sharma

**Affiliations:** 1 Internal Medicine, Jagadguru Jayadeva Murugarajendra (JJM) Medical College, Davangere, IND

**Keywords:** covid-19, c-reactive protein, red cell distribution width, platelet distribution width, prognosis

## Abstract

Introduction

Cytokine storm is central in the pathobiology of Coronavirus disease 2019 (COVID-19). The pro-inflammatory state and hypoxia disrupt erythropoiesis leading to alterations in red cell distribution width (RDW) and hematocrit. Platelet production increases alongside its destruction, inviting newly formed immature platelets into the circulation. Thus, the platelet distribution width (PDW) and mean platelet volume (MPV) are also affected. The study's objective is to analyze these indices and C-reactive protein (CRP) to elucidate prognostic insights in COVID-19 patients at the time of admission.

Methodology

This study was a retrospective cross-sectional study conducted at Chigateri General Hospital, attached to JJM Medical College, Davangere, over two months, July and August of 2020. Patients falling under categories B and C according to the interim guidelines issued by the Ministry of Health and Family Welfare, Government of India were enrolled in this study. Patients requiring mechanical ventilation and those with a prior diagnosis of malignancy were excepted from the study.

Results

The study population comprised a total of hundred patients. Seventy-five patients survived the disease and were discharged; twenty-five patients succumbed to the viral illness. The mean age of survivors (43.0 +/- 13.6 years) was significantly lesser than that of non-survivors (59.1 +/- 11.5 years) (p <0.001). RDW was significantly different among survivors (p=0.002); PDW and CRP were lower among the deceased (p=0.05 and p=0.10, respectively). Cut off values for RDW as 15%, CRP as 67 mg/l, and PDW as 17% were significantly associated with mortality. Hematocrit and MPV were not significantly associated with mortality. RDW has a sensitivity of 92% and a negative predictive value of 95% in predicting mortality.

Discussion

RDW showed a significant association with increased mortality. Impaired cell-mediated immunity at the onset of infection is responsible for rapid progression to moderate or even severe COVID disease. Since the investigations in our study were ordered at the time of admission, it may lead us to believe that higher RDW is associated with a better patient outcome.

Lower C-reactive protein levels are associated with higher mortality. CRP is a non-specific marker for inflammation. Raised CRP is customarily an indicator of acute inflammation. Notwithstanding, the raised CRP may be an indicator of baseline immune response in early COVID infection.

High PDW shows a significant association with increased mortality. The pathobiology of change in platelet indices in COVID-19 patients is presumably multifactorial: infection of the bone marrow; autoimmune platelet destruction; platelet sequestration.

Conclusion

Red cell distribution width, platelet distribution width, and C-reactive protein are useful early predictive markers of mortality in COVID-19. Although serial investigations would provide a better picture, these indices at admission can gauge the clinical outcome early in the disease. As there is still a lot to be understood about the natural history of COVID-19, our study aims to propose relatively inexpensive indices of mortality that can aid efficient management.

## Introduction

In December 2019, a new illness, caused by a novel severe acute respiratory syndrome coronavirus 2 (SARS-CoV-2), broke out in Wuhan city, China. In January 2020, the World Health Organization declared the outbreak a Public Health Emergency of International Concern [1].

Multifactorial causation is responsible for the relationship between mean platelet volume and Coronavirus disease 2019 (COVID-19). In a study conducted by Güçlü et al., there was an increase in mortality by 1.76 times for every one unit increase in mean platelet volume [2]. Bone marrow is infected, which leads to thrombocytopenia. There is a destruction of platelets by the immune system. Lastly, more platelets get consumed due to accumulation in the lungs. Platelet count decrease leads to an increase in platelet production. There is an increased production of young platelets, which are functionally more active than older platelets. All these factors lead to an increase in the mean platelet volume. Mean platelet volume can function as a simple, economical, quick, and widely available laboratory parameter that recognizes the severe presentation of COVID-19 [2]. 

Platelet distribution width (PDW) reflects the variation in the size of platelets. PDW increases when platelet destruction increases and there are variations in the size of newly formed immature platelets [3]. Increased cytokine release and inflammation lead to higher platelet production and increased platelet destruction. SARS-CoV-2 utilizes its spike protein to enter host cells by binding to angiotensin-converting enzyme 2 (ACE2) on the host cell membrane. Transmembrane protease serine 2, a serine protease, proteolytically cleaves and activates the spike protein to facilitate SARS-CoV-2 virus-cell membrane fusions. The spike protein potentiates thrombus formation. Coagulation factors are released, inflammatory cytokines are secreted, and leukocyte platelet aggregates are formed [4].

Red cell distribution width (RDW) conveys the degree of anisocytosis among red blood cells. Anisocytosis is a hugely inflammation dependent process. Many of the proinflammatory cytokines like TNF-α and interleukin-1 decrease erythropoietin production during cytokine storm. Additionally, hypoxia induces disruption of erythropoiesis in COVID-19. Super-added infections are quite common in COVID-19, therefore increasing sepsis. RDW plays a considerable role even in sepsis. Hematological analyzers automatically generate RDW and can hence be ordered multiple times per day [5]. Also, RDW can significantly predict mortality even after discharge from the intensive care unit [6].

The study's objective is to simultaneously assess and provide insights regarding several hematological indices in the mortality of COVID-19 patients at the time of admission.

## Materials and methods

This study was a retrospective cross-sectional study conducted at Chigateri General Hospital, attached to JJM Medical College, Davangere, over two months, July and August of 2020. Clearance was taken from the Institutional Ethics Committee to begin the study.

**Figure 1:**
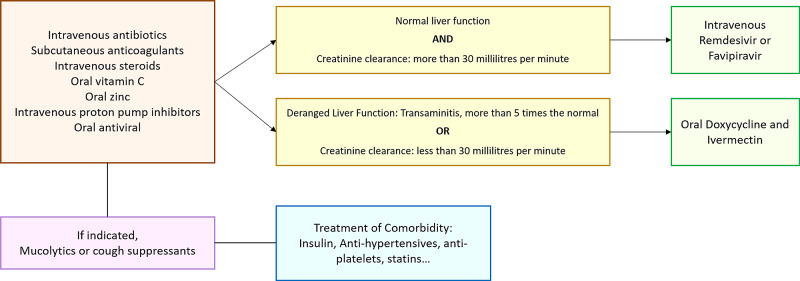
Treatment plan of all patients admitted under categories B and C in accordance with the interim guidelines issued by the Ministry of Health and Family Welfare, Government of India

Patients with a confirmed diagnosis of SARS-COV-2 infection, positive real-time polymerase chain reaction (RT-PCR) for viral ribonucleic acid (RNA) were included in the study. Only patients falling under categories B and C according to the interim guidelines issued by the Ministry of Health and Family Welfare, Government of India were enrolled in this study [7]. Patients requiring mechanical ventilation and those with a prior diagnosis of malignancy were excepted from the study. Tocilizumab, pirfenidone, azathioprine, and cyclophosphamide were not administered to any of the patients. Figure 1 presents the treatment plan followed for all patients included in this study.

The laboratory records and clinical data of the patients were accessed and analyzed on IBM Statistical Package for the Social Sciences (SPSS) Statistics for Windows, version 27 (IBM Corp., Armonk, NY). Hematocrit (HCT), co-efficient of variation of the red cell distribution width (RDW-CV), C-reactive protein (CRP), co-efficient of variation of the platelet distribution width (PDW), and the mean platelet volume (MPV) were analyzed for their ability to predict and prognosticate the clinical outcome.

The demographic data has been depicted as descriptive statistics. The unpaired t-test was employed to compare the mean between two groups of data. Mann-Whitney U test was employed to compare the mean of two sets of non-parametric data. The chi-square test was used for categorical data. Odd's ratio was used to calculate the odds of occurrence of mortality using these indices. Diagnostic validity tests and receiver operator characteristic curves were applied to analyze and contrast the different indices. A p-value of 0.05 or less was deemed statistically significant.

## Results

The study population comprised a total of 100 patients. Seventy-five patients survived the disease and were discharged; 25 patients succumbed to the viral illness. The study population's mean age was 47.1 ± 14.8 years, ranging from 20 years to 78 years. Of the 100 participants, 57 were males, and 43 were females.

The mean age of patients who survived the disease was 43 years, significantly lesser than that of non-survivors (59.1 years), with a p-value of <0.001 (Table 1).

**Table 1:** Descriptive information on study subjects HS: highly significant; NS: not significant; SD: standard deviation

Number of cases		All cases	Non-survivors	Survivors	Non-survivors vs survivors	
		100	25	75		
Age (years)	Mean ± SD	47.1 + 14.8	59.1 + 11.5	43.0 + 13.6	t = 5.31	P < 0.001, HS
	Range	20–78 yrs	40–78 yrs	20–71 yrs		
Sex	Male	57	13	44	X 2 = 0.34,	P = 0.56, NS
	Female	43	12	31		

The various hematological indices investigated in the study have been shown in Table 2.

**Table 2:** Descriptive statistics on test measurements HCT: hematocrit; RDW-CV: co-efficient of variation of the red cell distribution width; MPV: mean platelet volume; CRP: C-reactive protein; PDW: platelet distribution width; SD: standard deviation

Parameter	Normal range	Mean + SD	Median	Minimum	Maximum
Hemoglobin (g/dl)	12–16	12.2 + 2.44	12.3	5.6	15.7
Platelets (/cumm)	150,000 to 450,000	22849 + 9646.2	22200	2300	467000
HCT (%)	38–50	38.1 + 7.4	39.5	13.7	49.7
RDW-CV (%)	11.8–16.1	14.6 + 2.9	14.1	7.1	22
MPV (fl)	7.5–12.0	8.68 + 1.43	8.20	6.9	12.6
CRP (mg/l)	0–10	63.5 + 40.2	64.25	0.6	292.7
PDW (%)	15–17	16.7 + 2.7	17.4	11.2	21.5

Table 3 presents the different indices in a comparison between survivors and non-survivors. RDW was significantly higher among the patients who survived the disease with a p-value of 0.002 as against the non-survivors. PDW and CRP were lower among the deceased, and the strength of the associations were p=0.05 and 0.10, respectively.

**Table 3:** Comparison of test parameters between non-survivors and survivors HCT: hematocrit; RDW: red cell distribution width; MPV: mean platelet volume; CRP: C-reactive protein; PDW: platelet distribution width; NS: not significant; S: significant; SD: standard deviation The unpaired t-test has been used in the analysis of data that followed a normal distribution (HCT, MPV, and PDW). RDW and CRP were analyzed using the Mann-Whitney U test.

Parameter	Non-survivors (n=25)		Survivors (n=75)		Non-survivors vs survivors	
	Mean	SD	Mean	SD	t	p-value
HCT	38.4	6.6	38.0	7.7	0.23	0.82, NS
RDW	13.9	2.2	14.8	3.0	-	0.002, S
MPV	8.47	1.42	8.75	1.44	0.84	0.40, NS
CRP	56.04	28.19	66.01	43.34	-	0.10, S
PDW	17.58	2.84	16.37	2.59	1.96	0.05, S

Table 4 presents the correlation of RDW, PDW, and CRP concerning the clinical outcome. Cut off values for RDW as 15%, CRP as 67 mg/l, and PDW as 17% were significantly associated with mortality. Haematocrit and MPV were not significantly associated with mortality.

**Table 4:** Index-wise distribution of cases and their significance in differentiating the final outcome RDW: red cell distribution width; CRP: C-reactive protein; PDW: platelet distribution width; S: significant

Test parameter	Cut-off value	Non-survivors (n=25)		Survivors (n=75)		Non-survivors vs survivors		Odds ratio (95% CI)
		No.	%	No.	%	X ²	P-value	
RDW	≤ 15.0	23	92.0	40	53.3	12.02	0.001, S	10.1 (2.2-45.8)
	> 15.0	2	8.0	35	46.7			
CRP	≤ 67.0	19	76.0	37	49.3	5.41	0.02, S	3.3 (1.2-9.1)
	> 67.0	6	24.0	38	50.7			
PDW	≤ 17.0	21	84.0	42	56.0	6.31	0.012, S	4.1(1.3 - 13.2)
	> 17.0	4	16.0	33	44.0			

**Figure 2:**
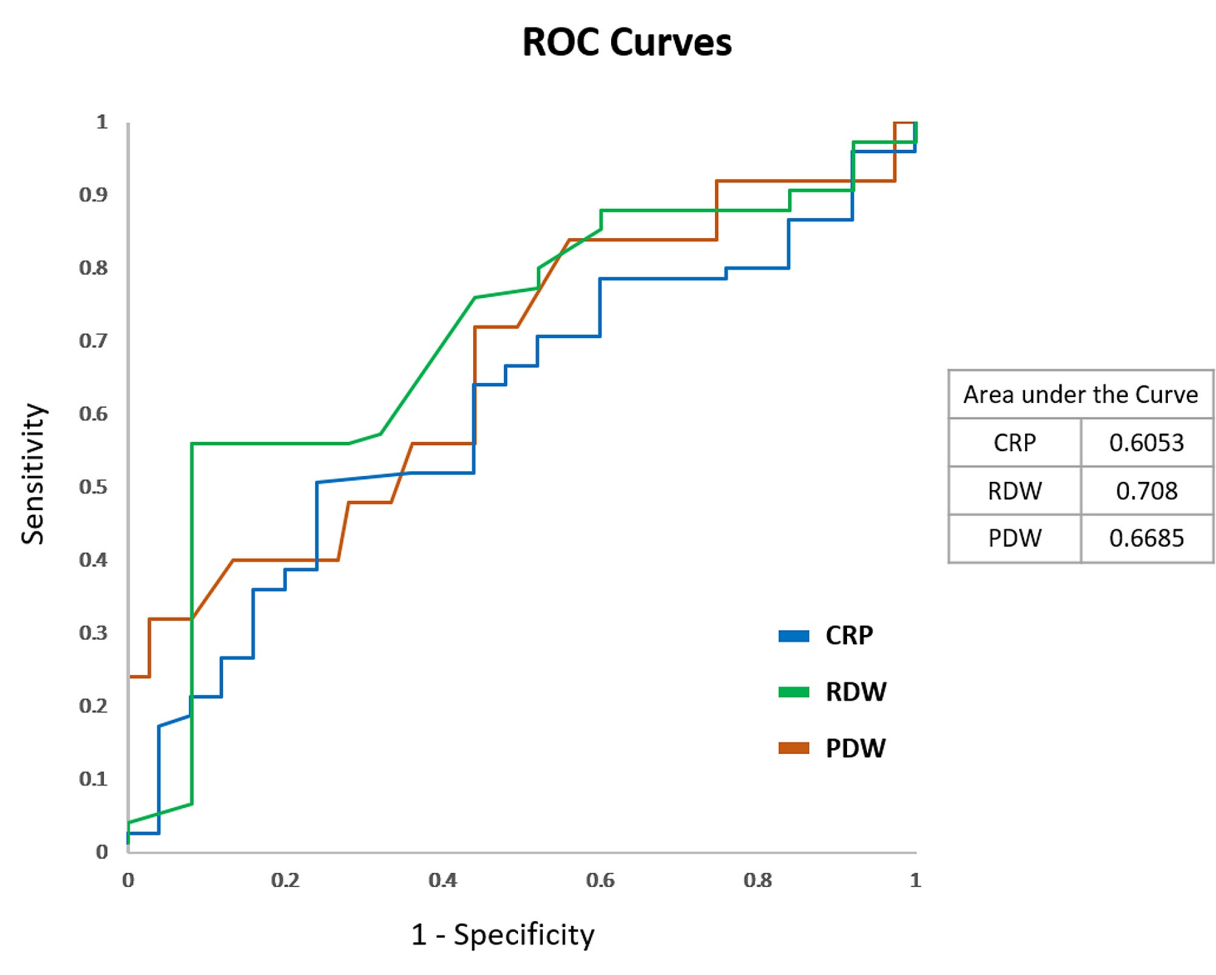
ROC curve comparing the diagnostic validity of RDW, PDW, and CRP in the prediction of mortality among COVID-19 patients ROC: receiver operating characteristic; RDW: red cell distribution width; CRP: C-reactive protein; PDW: platelet distribution width

The diagnostic validity of RDW, PDW, and CRP at the abovementioned cut-off values have been presented in Table 5. RDW has a sensitivity of 92% and a negative predictive value of 95% in predicting mortality. Figure 2 compares the diagnostic validity of RDW, PDW, and CRP in the prediction of mortality.

**Table 5:** Diagnostic validity tests for predicting mortality using various significant parameters PPV: positive predictive value; NPV: negative predictive value; RDW: red cell distribution width; CRP: C-reactive protein; PDW: platelet distribution width

	RDW	CRP	PDW
	≤ 15.0	≤ 67.0	≤ 17.0
Sensitivity	92%	76%	84%
Specificity	47%	51%	44%
PPV	37%	34%	34%
NPV	95%	87%	89%
Accuracy	58%	57%	54%

## Discussion

RDW showed a significant association with increased mortality. With a cut-off value as 15 mg/dl, RDW of lower than 15% was significantly associated with increased mortality (X ²= 12.02, p = 0.001). RDW also has a high sensitivity of 92% and a high negative predictive value of 95% in predicting adverse outcomes. This observation disagrees with a study done by Foy et al., which found that RDW higher than 14.5% was associated with higher mortality [8]. Also, a study by Gong et al. ascertained a similar result. Uncontrolled immune activation in COVID-19 can result in increased red cell turnover, which can raise RDW. Various cytokines like IL-1 and TNF alpha can raise erythropoietin secretion, raising RDW [9]. Conversely, Sharma et al. concluded that there was no significant association between RDW and mortality [10]. This ambiguity shows that the relationship between RDW and mortality is not consistent, and further investigation into the same is warranted. 

Impaired cell-mediated immunity at the onset of infection is responsible for rapid progression to moderate or even severe COVID disease. Since the investigations in our study were ordered at the time of admission, it may lead us to believe that higher RDW is associated with a better patient outcome. However, the modestly raised RDW is within normal limits and may indicate baseline immune response in early infection. The importance of robust cell-mediated immunity in preventing severe COVID has adequately been described in the literature [11]. 

Lower C-reactive protein levels are associated with higher mortality, but the association's significance was defined at smaller confidence intervals (p < 0.10). CRP lower than 67 mg/dl was associated with higher mortality (X² = 5.41, p = 0.02). CRP also has a high negative predictive value of 87% and a high sensitivity of 76%. In a study by Wang, the size of the pulmonary lesion and CRP levels showed a positive correlation [12]. Findings in our study were conflicting with this. CRP is a non-specific marker for inflammation. Raised CRP is customarily an indicator of acute inflammation [13]. Notwithstanding, the raised CRP may be an indicator of baseline immune response in early COVID infection. An appropriate brisk immune response in the early stages of infection could decrease viral load and, by extension, decrease illness severity. Since the patients included in the study were in the early stages of infection, presenting within four days of symptoms, low CRP could indicate a weak baseline immune response, thereby resulting in a worse clinical outcome [11]. 

High PDW shows a significant association with increased mortality. A cut-off value of 17% was significantly associated with increased mortality (X² = 6.31, p = 0.012). PDW also has a high sensitivity of 84% and a high negative predictive value of 89%. This finding concurs with Güçlü et al. and Yun et al. [2,14]. The pathobiology of change in platelet indices in COVID-19 patients is presumably multifactorial. The following three hypotheses related to platelet count and structure are proposed in COVID-19:

1. As with other coronaviruses, thrombocytopenia is perhaps due to infection of the bone marrow.

2. SARS-CoV-2 is involved in systemic immune regulation, which may cause autoimmune platelet destruction [11,13].

3. Platelet sequestration in the lungs in response to alveolar damage may be responsible for the altered finding.

Briefly, platelet production increases while platelet counts decrease [13].

Limitations

A limitation of this study is that financial and logistical constraints came in the way of conducting serial investigations. The size of the sample was small and, larger studies are necessary to validate the findings. Also, follow-up of patients after discharge could not be done.

## Conclusions

Red cell distribution width, platelet distribution width, and C-reactive protein are useful, inexpensive, and early predictive markers of mortality in COVID-19. Although serial investigations would provide a better picture, these indices at admission can gauge the clinical outcome early in the disease. As there is still a lot to be understood about the natural history of COVID-19, our study aims to propose relatively inexpensive indices of mortality that can aid efficient management.

## Appendices

**Table 6:** Confusion matrices used for prediction of mortality with RDW, CRP, and PDW RDW: red cell distribution width; CRP: C-reactive protein; PDW: platelet distribution width Cut-off values for predicting mortality: RDW-15%; CRP-67 mg/l; PDW-17%.

Confusion matrix-RDW		Clinical outcome	
		Non-survivors	Survivors
Red cell distribution width	< 15%	23	40
	> 15%	2	35
Confusion matrix-CRP			
C reactive protein	< 67 mg/l	19	37
	> 67 mg/l	6	38
Confusion matrix-PDW			
Platelet distribution width	> 17%	21	42
	< 17%	4	33

**Table 7:** The data sheet containing details of all the patients in this study WBC: white blood cell; f: female; m: male; RDW: red cell distribution width; MPV: mean platelet volume; CRP: C-reactive protein; PDW: platelet distribution width

Patient no.	Age	Sex	Hemoglobin (g/dl)	RBC (million/cumm)	Hematocrit	WBC (/cumm)	Platelets (/cumm)	RDW	PDW	MPV	PDW/MPV	Survival group	CRP
1	26	f	11.80	4.15	35.6	7900	21100	11.8	19.6	12.6	1.556	Survivors	1.1
2	43	m	14.30	4.09	41.6	7900	19000	17	13.1	8.2	1.598	Survivors	2
3	68	f	11.70	4.3	37	11100	25900	13.9	17.4	10.1	1.723	Non-survivors	57.5
4	48	f	6.00	4.15	29	21200	17500	11.6	21.5	6.9	3.116	Non-survivors	27.1
5	35	f	6.60	4.15	23	21200	11600	2.1	15.7	8.2	1.915	Survivors	62.0
6	53	f	11.00	3.98	35.8	5000	10400	15.4	17.1	7.8	2.192	Survivors	73.5
7	65	m	14.50	5.09	46.1	12000	31400	13.1	19.7	7	2.814	Non-survivors	64.9
8	47	m	13.00	4.72	40.3	25400	35600	15.7	12	12	1.000	Survivors	58.4
9	35	m	6.60	4.15	23	21200	11600	2.1	15.7	8.2	1.915	Survivors	67.9
10	71	m	14.40	4.7	43	15600	45900	14	19.5	8.2	2.378	Survivors	74.7
11	51	m	12.90	4.69	40	19000	10300	15.3	18.9	8.6	2.198	Survivors	67.7
12	53	f	11.00	3.98	35.8	5000	2300	15.00	17.1	7.8	2.192	Survivors	10.9
13	42	m	13.60	4.82	42.1	8600	24100	14.1	17	8.2	2.073	Survivors	87.6
14	22	m	15.50	5.07	44.7	6400	31300	11	11.9	10.3	1.155	Survivors	2.1
15	27	m	5.60	2.6	27	10400	19000	20.3	17.2	8.4	2.048	Survivors	76.3
16	65	m	12.30	4.94	39	14400	38300	15	18.3	7.4	2.473	Survivors	67.0
17	21	f	11.30	3.96	36.5	6400	16300	13.8	12.2	8.4	1.452	Survivors	8.7
18	73	m	14.30	5.49	45.7	4400	14300	12.9	17.9	7.2	2.486	Non-survivors	60.1
19	40	f	10.40	4	34.5	18000	25300	20	17.5	8.4	2.083	Non-survivors	123.1
20	45	m	13.90	4.21	41	5000	17100	10.4	20	10	2.000	Non-survivors	7.9
21	35	m	13.50	4.8	43	7500	10200	13.7	19	6.9	2.754	Survivors	74.3
22	73	m	14.30	5.49	45.7	4400	14300	12.9	17.9	7.2	2.486	Non-survivors	71.6
23	52	m	14.50	5.32	45	6800	27500	14	19.8	7.6	2.605	Survivors	74.9
24	28	f	14.10	4.53	42.2	7400	33500	16	13.5	7	1.929	Survivors	105.2
25	60	m	14.60	5.14	44.1	4700	14500	18	12.1	9.3	1.301	Survivors	4.7
26	39	m	8.70	3.76	28.5	5400	16400	15	19.1	8	2.388	Survivors	59.3
27	40	m	12.30	4.7	40.6	8600	21200	20	17.6	8.4	2.095	Survivors	72.1
28	42	f	10.10	3.46	30.3	14600	39000	13.9	18.4	7.4	2.486	Survivors	67.1
29	37	f	10.00	4.9	33.3	4900	27200	17.7	16.8	8.2	2.049	Survivors	55.8
30	60	m	10.40	4.7	35.3	14600	14200	15.9	19.1	8	2.388	Survivors	68.6
31	64	m	15.70	5.36	48	10000	28600	13.7	20.2	7	2.886	Non-survivors	74.6
32	70	m	10.80	7.8	35.7	14100	23700	16	16	10	1.600	Survivors	59.6
33	55	f	10.30	3.53	31	16400	21600	13.5	18.4	7.4	2.486	Non-survivors	70.5
34	25	m	11.40	4.11	37	8300	30400	16.7	18.6	8.2	2.268	Survivors	121.1
35	21	m	11.30	3.96	36.5	6400	16300	13.8	12.2	8.4	1.452	Survivors	58.5
36	49	m	15.10	4.74	30.1	3800	33000	10.9	11.2	7	1.600	Survivors	2.2
37	27	f	5.60	2.6	29	10400	19000	20.3	17.2	8.4	2.048	Survivors	60.0
38	78	f	9.00	3.77	29.4	7200	29200	14.9	19.1	7.4	2.581	Non-survivors	55.9
39	60	f	11.90	4.62	37.9	10200	23500	14	17	10.2	1.373	Non-survivors	1.9
40	30	f	14.70	5.42	45.8	8500	22200	14	17.9	9.3	1.925	Survivors	59.9
41	35	m	13.50	4.8	43	7500	2800	13.7	19	6.9	2.754	Survivors	123.4
42	40	f	10.40	4	34.5	18000	25300	20	17.5	8.4	2.083	Non-survivors	49.4
43	52	m	14.50	5.32	45	6800	27500	14	19.8	7.6	2.605	Survivors	63.2
44	50	m	13.90	4.9	44	4300	15600	14	12.1	12.6	0.960	Survivors	71.3
45	50	m	14.80	5.73	48	4500	17800	14	17	9.3	1.828	Survivors	70.1
46	50	m	13.90	4.9	44	4300	15600	14	12.1	12.6	0.960	Survivors	77.9
47	37	m	10.00	4.9	33.3	4900	27200	17.7	16.8	8.2	2.049	Survivors	112.4
48	43	m	14.00	4.7	44.8	8300	7500	15.1	18.2	7.8	2.333	Survivors	58.1
49	46	f	10.60	4.2	40	800	30800	15.1	18.5	8.6	2.151	Survivors	72.8
50	30	m	14.70	5.42	45.8	8500	22200	14	17.9	9.3	1.925	Survivors	67.5
51	51	m	12.90	4.69	40	19000	11100	15.3	18.9	8.6	2.198	Survivors	63.9
52	44	f	11.50	4.3	36	4200	10000	15	16.2	10.4	1.558	Survivors	67.5
53	60	f	11.90	4.62	37.9	10200	23500	14	17	10.2	1.373	Non-survivors	77.3
54	56	m	7.70	2.85	24.5	6200	33000	15.2	13.5	10.3	1.311	Survivors	118
55	65	f	12.30	4.94	39	14400	38300	15	18.3	7.4	2.473	Survivors	118.2
56	52	f	12.20	4.32	38.1	13100	11300	14	15	10	1.500	Survivors	60.9
57	52	m	12.20	4.32	38.1	13100	2700	14	15	10	1.500	Survivors	4.8
58	68	f	11.70	4.3	37	11100	25900	13.9	17.4	10.1	1.723	Non-survivors	55.9
59	39	f	8.70	3.76	28.5	5400	16400	15	19.1	8	2.388	Survivors	57.7
60	50	f	14.80	5.73	48	4500	17800	14	17	9.3	1.828	Survivors	73.0
61	71	m	14.40	4.7	43	15600	45900	14	19.5	8.2	2.378	Survivors	292.7
62	66	m	14.50	5	40	31400	13100	14.3	11.7	10	1.170	Non-survivors	67.0
63	53	m	13.50	4.65	42	7200	22200	15.4	15	8	1.875	Survivors	102.9
64	32	m	13.80	4.7	43.9	8800	46700	14	18.9	7.2	2.625	Survivors	13.5
65	42	m	13.60	4.82	42.1	8600	24100	14.1	17	8.2	2.073	Non-survivors	59.8
66	46	f	10.60	4.2	3.7	800	30800	15.4	18.5	8.6	2.151	Survivors	60.3
67	53	f	13.50	4.65	42	7200	22200	15.4	15	8	1.875	Survivors	24.7
68	46	f	11.70	4.63	37	30700	26100	20	15	11	1.364	Survivors	19.1
69	60	m	14.60	5.14	44.1	4700	14500	11	12.1	9.3	1.301	Survivors	3
70	48	m	6.00	4.15	25	21200	11400	11.6	21.5	6.9	3.116	Non-survivors	8.1
71	66	m	14.50	5	40	6000	13100	14	11.7	10	1.170	Non-survivors	67.0
72	65	m	14.50	5.09	46.1	12000	31400	13.1	19.7	7	2.814	Non-survivors	64.3
73	25	f	11.40	4.11	37	8300	30400	16.7	17	10.4	1.635	Survivors	69.3
74	25	f	11.40	4.11	37	8300	30400	16.7	18.6	8.2	2.268	Survivors	119.9
75	65	m	11.10	4	34	11000	25100	13.7	17.8	9.5	1.874	Survivors	118.6
76	46	f	13.90	4.88	43.6	6800	14000	15.6	16.7	8.2	2.037	Survivors	59.5
77	46	m	13.90	4.88	43.6	6800	14000	15.2	16.7	8.2	2.037	Survivors	71.1
78	32	f	13.80	4.7	43.9	8800	46700	14	18.9	7.2	2.625	Survivors	117.4
79	38	m	15.20	5.41	49.7	5400	15100	13.6	13.1	8	1.638	Survivors	93.4
80	20	f	11.00	4	35.4	9800	32500	17.3	17.7	7	2.529	Survivors	58.5
81	78	f	9.00	3.77	29.4	7200	29200	14.9	19.1	7.4	2.581	Non-survivors	1.8
82	25	f	11.40	4.11	37	8300	30400	16.7	17	10.4	1.635	Survivors	61.6
83	26	f	11.80	4.15	35.6	7900	21100	11.8	19.6	12.6	1.556	Survivors	121.3
84	64	m	15.70	5.36	48	10000	28600	13.7	20.2	7	2.886	Non-survivors	59.0
85	22	m	15.50	5.07	44.7	6400	31300	11	11.9	10.3	1.155	Survivors	2.3
86	60	f	10.40	4.7	35.3	14600	14200	15.9	19.1	8	2.388	Survivors	76.6
87	20	f	11.00	4	35.4	9800	32500	17.3	17.7	7	2.529	Survivors	64.2
88	56	f	13.60	4.52	41.7	8800	30800	14.4	13.9	10.2	1.363	Non-survivors	59.2
89	45	m	13.90	4.21	41	5000	17100	10.4	20	10	2.000	Non-survivors	67.0
90	56	m	7.70	2.85	44.5	6200	33000	15.3	13.5	10.3	1.311	Survivors	67.8
91	38	m	15.20	5.41	49.7	5400	15000	13.6	13.1	8	1.638	Survivors	120.4
92	45	f	11.70	4.9	37	11000	21700	15.9	18	8.8	2.045	Survivors	1.1
93	56	m	13.60	4.52	41.7	8800	30800	14.4	13.9	10.2	1.363	Non-survivors	56.6
94	49	m	15.10	4.74	15.1	3800	33000	10.9	11.2	7	1.600	Survivors	72.4
95	43	f	14.30	4.09	41.6	7900	19000	19	13.1	8.2	1.598	Survivors	73.3
96	40	m	12.30	4.7	40.6	8600	21200	22	17.6	8.4	2.095	Survivors	0.6
97	44	m	11.50	4.3	36	4200	10000	15	16.2	10.4	1.558	Survivors	62.1
98	28	m	14.10	4.53	42.2	7400	33500	15	13.5	7	1.929	Survivors	57.5
99	65	f	11.10	4	34	11000	25100	13.7	17.8	9.5	1.874	Survivors	62.1
100	55	f	10.30	3.53	31	16400	21600	13.5	18.4	7.4	2.486	Non-survivors	93.4
